# Understanding the role of Fas-Fas ligand system in bone

**DOI:** 10.1186/ar3575

**Published:** 2012-02-09

**Authors:** Ana Marusic, Natasa Kovacic, Ivan Kresimir Lukic, Vedran Katavic, Danka Grcevic

**Affiliations:** 1 Department of Anatomy and Department of Research in Biomedicine and Health, University of Split School of Medicine, Split, Croatia; 2 Department of Anatomy, University of Zagreb School of Medicine, Zagreb, Croatia; 3 Partek Inc, Zagreb, Croatia; 4 Department of Physiology and Immunology, University of Zagreb School of Medicine, Zagreb, Croatia

## 

Fas ligand (CD 178) and its receptor Fas (CD 95) are members of the TNF superfamily of ligands and receptors involved in the activation of apoptosis. Our research group demonstrated that Fas and Fas ligand were expressed during osteoblast and osteoclast differentiation, and their expression may be modified by various cytokines. The lack of functional Fas signaling in murine models leads to altered endochondral ossification, increase of the bone mass in adult mice, and resistance to ovariectomy-induced bone loss. We also showed that mice with a Fas gene knockout lose less bone during antigen-induced arthritis. These changes seem to be, at least in part, mediated by increased expression of osteoprotegerin (OPG), another member of the TNF superfamily, which acts as a decoy receptor for receptor activator for nuclear factor κB (RANK) ligand (RANKL). The bone phenotype of mice lacking Fas signaling may be related to the immunological disturbance rather than intrinsic bone disorder. To address this question at molecular level, we performed a set of parabiotic experiments in mice with non-functional Fas ligand mutation (gld mice). Mice were kept in parabiosis for 1 to 4 weeks, and for 2 weeks after separation from 4-week parabiosis. We also analyzed OPG levels in the peripheral blood of patients with autoimmune lymphoproliferative syndrome (ALPS). Joined circulation between gld and wild-type mice led to increased expression of bone protective OPG in the wild-type animal, both at the gene and protein level at 4 weeks of parabiosis. This effect was sustained even after the separation of parabiotic mice. At the same time, double-negative T lymphocytes transferred from gld into wild-type member of a parabiotic pair rapidly vanished from the periphery of both gld and control mice in parabiosis. Patients with ALPS had increased OPG mRNA level in peripheral blood mononuclear cells, as assessed by real-time PCR, in comparison to age- and sex-matched controls. These findings show that bone and immune changes are uncoupled during Fas ligand deficiency. Under the assumption that OPG also acts as a molecular brake in the immune system, downregulation of OPG in gld mice during parabiosis with wild-type mice could be considered as a molecular marker of remission. Increased expression of OPG in children with ALPS leads to the hypothesis that a similar mechanism might be at play in humans.

